# Supplementary material for: Causal Relationship between Aging and Anorexia Nervosa: A White-Matter-Microstructure-Mediated Mendelian Randomization Analysis
Source: Biomedicines. 2024 Aug 16;12(8):1874. doi: 10.3390/biomedicines12081874 (PMC11351342; doi:10.3390/biomedicines12081874)
Supplement: Supplementary file 1 [file biomedicines-12-01874-s001.zip › Supplementary Figure/Figure S4/Singe SNP plot of MR analysis for TL to ubm-b-1476.pdf]

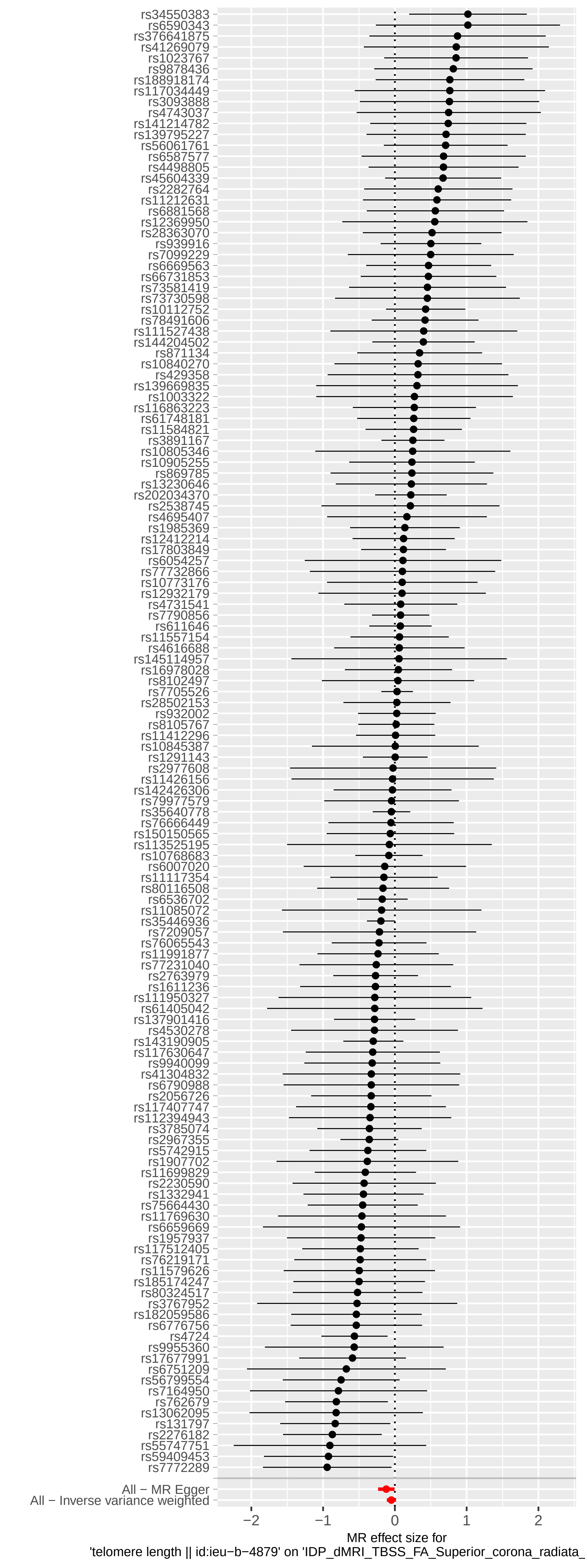
rs34550383
rs6590343
rs376641875
rs41269079
rs1023767
rs9878436
rs188918174
rs117034449
rs3093888
rs4743037
rs141214782
rs139795227
rs56061761
rs6587577
rs4498805
rs45604339
rs2282764
rs11212631
rs6881568
rs12369950
rs28363070
rs939916
rs7099229
rs6669563
rs66731853
rs73581419
rs73730598
rs10112752
rs78491606
rs111527438
rs144204502
rs871134
rs10840270
rs429358
rs139669835
rs1003322
rs116863223
rs61748181
rs11584821
rs3891167
rs10805346
rs10905255
rs869785
rs13230646
rs202034370
rs2538745
rs4695407
rs1985369
rs12412214
rs17803849
rs6054257
rs77732866
rs10773176
rs12932179
rs4731541
rs7790856
rs611646
rs11557154
rs4616688
rs145114957
rs16978028
rs8102497
rs7705526
rs28502153
rs932002
rs8105767
rs11412296
rs10845387
rs1291143
rs2977608
rs11426156
rs142426306
rs79977579
rs35640778
rs76666449
rs150150565
rs113525195
rs10768683
rs6007020
rs11117354
rs80116508
rs6536702
rs11085072
rs35446936
rs7209057
rs76065543
rs11991877
rs77231040
rs2763979
rs1611236
rs111950327
rs61405042
rs137901416
rs4530278
rs143190905
rs117630647
rs9940099
rs41304832
rs6790988
rs2056726
rs117407747
rs112394943
rs3785074
rs2967355
rs5742915
rs1907702
rs11699829
rs2230590
rs1332941
rs75664430
rs11769630
rs6659669
rs1957937
rs117512405
rs76219171
rs11579626
rs185174247
rs80324517
rs3767952
rs182059586
rs6776756
rs4724
rs9955360
rs17677991
rs6751209
rs56799554
rs7164950
rs762679
rs13062095
rs131797
rs2276182
rs55747751
rs59409453
rs7772289
All − MR Egger
All − Inverse variance weighted
−2
−1
0
1
2
MR effect size for
'telomere length || id:ieu−b−4879' on 'IDP_dMRI_TBSS_FA_Superior_corona_radiata_
